# Clinical and biological relevance of the transcriptomic‐based prostate cancer metastasis subtypes MetA‐C

**DOI:** 10.1002/1878-0261.13158

**Published:** 2021-12-27

**Authors:** Elin Thysell, Linda Köhn, Julius Semenas, Helena Järemo, Eva Freyhult, Marie Lundholm, Camilla Thellenberg Karlsson, Jan‐Erik Damber, Anders Widmark, Sead Crnalic, Andreas Josefsson, Karin Welén, Rolf J. A. Nilsson, Anders Bergh, Pernilla Wikström

**Affiliations:** ^1^ Department of Medical Biosciences, Pathology Umeå University Sweden; ^2^ Department of Radiation Sciences, Oncology Umeå University Sweden; ^3^ Department of Cell and Molecular Biology National Bioinformatics Infrastructure Sweden Science for Life Laboratory Uppsala University Sweden; ^4^ Department of Urology Sahlgrenska Center for Cancer Research Institute of Clinical Sciences Sahlgrenska Academy University of Gothenburg Sweden; ^5^ Department of Surgery and Perioperative Sciences, Orthopedics Umeå University Sweden; ^6^ Department of Surgery and Perioperative Sciences, Urology & Andrology Umeå University Sweden; ^7^ Wallenberg Center for Molecular Medicine Umeå University Sweden

**Keywords:** MetA‐C, metastasis, prognosis, prostate cancer, subtypes, transcriptomic

## Abstract

To improve treatment of metastatic prostate cancer, the biology of metastases needs to be understood. We recently described three subtypes of prostate cancer bone metastases (MetA‐C), based on differential gene expression. The aim of this study was to verify the clinical relevance of these subtypes and to explore their biology and relations to genetic drivers. Freshly‐frozen metastasis samples were obtained as hormone‐naive (*n* = 17), short‐term castrated (*n* = 21), or castration‐resistant (*n* = 65) from a total of 67 patients. Previously published sequencing data from 573 metastasis samples were also analyzed. Through transcriptome profiling and sample classification based on a set of predefined MetA‐C‐differentiating genes, we found that most metastases were heterogeneous for the MetA‐C subtypes. Overall, MetA was the most common subtype, while MetB was significantly enriched in castration‐resistant samples and in liver metastases, and consistently associated with poor prognosis. By gene set enrichment analysis, the phenotype of MetA was described by high androgen response, protein secretion and adipogenesis, MetB by high cell cycle activity and DNA repair, and MetC by epithelial‐to‐mesenchymal transition and inflammation. The MetB subtype demonstrated single nucleotide variants of RB transcriptional corepressor 1 (*RB1*) and loss of 21 genes at chromosome 13, including *RB1*, but provided independent prognostic value to those genetic aberrations. In conclusion, a distinct set of gene transcripts can be used to classify prostate cancer metastases into the subtypes MetA‐C. The MetA‐C subtypes show diverse biology, organ tropism, and prognosis. The MetA‐C classification may be used independently, or in combination with genetic markers, primarily to identify MetB patients in need of complementary therapy to conventional androgen receptor‐targeting treatments.

## Graphical abstract

Prostate cancer metastases were recently classified into subtypes, namely, MetA, MetB, and MetC (MetA‐C), based on their transcriptomic profiles. This study verifies the clinical relevance of the MetA‐C subtype classification and demonstrates its association with patient prognosis and tumor biology in several separate cohorts. The MetA‐C classification may be used to identify MetB patients in need of complementary therapy to conventional androgen receptor‐targeting therapies.

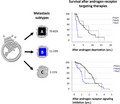

**Abbreviations**
ADT: androgen deprivation therapy
AR: androgen receptor
ARSI: androgen receptor signaling inhibitors
CNV: copy number variant
EMT: epithelial‐to‐mesenchymal transition
GSEA: gene set enrichment analysis
mCRPC: metastatic castration‐esistant prostate cancer
Met: metastasis
NEPC: neuroendocrine prostate cancer
NES: normalized enrichment scores
OPLS‐DA: orthogonal projections to latent structures discriminant analysis
PARP: poly‐ADP ribose polymerase
PC: prostate cancer
PCA: principal components analysis
PSA: prostate specific antigen
RMA: robust multi‐array average
SNV: single nucleotide variant

## 1 Introduction

Prostate cancer (PC) is the most common malignancy in men and a major cause of cancer mortality worldwide. Patients with lethal PC develop bone metastatic disease that is primarily treated with androgen deprivation therapy (ADT). In most cases, ADT reduces metastasis growth, but eventually metastatic castration‐resistant PC (mCRPC) develops. Several treatment strategies for mCRPC exist of which the majority aim at inhibiting androgen receptor (AR) signaling, while others include chemotherapy, immunotherapy, bone‐targeting therapies, and poly‐ADP ribose polymerase (PARP) inhibitors [1]. Patients show diverse responses to available therapies for metastatic PC, ranging from strong response to complete resistance, underlining the need for therapy‐predictive biomarkers and improved treatment strategies. To accomplish this, the tumor biology of metastatic PC needs be understood in more detail, particularly as the response to treatments could be site‐dependent and thus different in primary tumors vs. metastases [2].

Primary PC and its metastases demonstrate genomic heterogeneity, especially in the mCRPC stage [3, 4, 5, 6, 7, 8], but the prognostic and therapy‐predictive value of most DNA alterations remain unclear. Studies of metastatic samples from mCRPC patients have described *RB1* alteration as a marker for poor prognosis [7, 9], and the only genetic markers associated with poor survival after treatment with androgen receptor signaling inhibitors (ARSI) [7]. In parallel, multiple gene expression classifiers have been developed to differentiate indolent from progressive PC at diagnosis [10, 11, 12] and/or to highlight primary PC of different molecular subtypes [13, 14]. Single‐cell sequencing data further suggest that individual prostate tumors contain several subclones of tumor epithelial cells with different molecular characteristics [15, 16]. We have recently explored the transcriptome of PC bone metastases and identified three subtypes, termed MetA‐C, based on unsupervised cluster analysis of transcript levels [17]. The MetA‐C subtypes were further characterized by differences in morphology, phenotype, and patient outcome. The most common subtype, MetA, demonstrated high AR activity, while MetB and MetC both showed low AR activity. Accordingly, MetA patients had the most favorable outcome after ADT. In addition, MetB showed high cell cycle activity and DNA damage and, consequently, MetB patients had the worst prognosis. Based on our own collection of a validation cohort, and with clinical data published alongside RNA and DNA profiles in external data sets [5, 7, 9], the current study was performed to verify the clinical and biological relevance of the MetA‐C subtypes and, moreover, to explore the MetA‐C subtypes in relation to genetic drivers.

## 2 Materials and methods

### 2.1 Patient samples

Fresh‐frozen metastasis tissue samples were obtained from a total of 67 patients with bone metastatic PC (Table 1). The majority (*n* = 49) underwent surgery for metastatic spinal cord compression (Umeå University Hospital or Sahlgrenska University Hospital, 2003–2019). Core biopsies from the iliac crest or lymph node metastases were obtained from 16 and 2 patients, respectively. At the time for sampling, patients were either hormone‐naïve (*n* = 15), castration‐resistant (*n* = 42), or treated with ADT for a shorter period ranging between 1 day and 3 months (short‐term castrated, *n* = 10). From some patients, replicate samples were taken from the same metastasis site either at one time‐point (*n* = 14) or at different time‐points with additional therapies given in‐between (*n* = 6), and the study totally included 103 metastasis samples (for details, Table S1). When replicate metastasis samples were obtained from a patient at one time‐point, mean MetA‐C values were calculated and the patient was represented once in analyses related to clinical characteristics. When repetitive metastasis samples were collected from a patient at different occasions with additional therapy given in‐between, the patient was represented twice in analyses related to clinical characteristics. The study was approved by the regional ethic review boards in Umeå (Dnr 03‐158, Dnr 04‐26M, 2007‐08‐24) and Gothenburg (Dnr 455‐11). Patients gave their verbal and written consents.

**Table 1:** Clinical characteristics of 67 patients with bone metastatic prostate cancer from whom metastatic tissue were sampled and profiled by Clariom D array analysis. Continuous variables given as median (25th; 75th percentiles).

	Median (25th; 75th percentiles)
Age diagnosis (years)	70 (66–77)
Age metastasis surgery (years)	74 (70–78)
Serum PSA diagnosis (ng·mL^−1^)	110 (20–870)
Serum PSA metastasis surgery (ng·mL^−1^)	150 (39–910)

	*N* (%)
Gleason score at diagnosis	
6	5 (8%)
7	13 (19%)
8–10	28 (42%)
Not available	21 (31%)
Primary treatment	
Radical prostatectomy	9 (13%)
Radiotherapy	8 (12%)
Castration	50 (75%)
Castration therapy^a^	
None (hormone‐naïve)	15 (22%)
Short‐term^b^	10 (15%)
Long‐term	42 (63%)
Treatment for CRPC	
Bicalutamide	26 (39%)
Chemotherapy	13 (19%)
Abiraterone acetate	6 (9%)
Enzalutamide	5 (8%)
Ra223	3 (5%)
Zoledronic acid/Denosumab	5 (7%)

^a^ Castration therapies given prior to collection of metastasis tissue samples included surgical ablation, LHRH/GnRH agonist therapy, or bicalutamide treatment.

^b^ Castration therapy for 1 day to 3 months before metastasis tissue sampling.

The study also included analysis of previously published RNA and whole‐exome sequencing data from external patient cohorts, obtained by analysis of metastasis samples from different organs in patients with mCRPC, here referred to as the Quigley [5] and the Abida [7] cohorts. Corresponding patient data for the Quigley cohort (*n* = 101) were available in [9], including serum levels of the prostate specific antigen (PSA) and time for overall survival from diagnosis of mCRPC. In the Abida cohort, RNA data were available from a total of 332 metastasis samples, delivered as two overlapping sets based on different sequencing libraries (polyA library, *n* = 266, and capture, *n* = 208). Complementary DNA data were available for 331 metastases and clinical data were available for subsets of patients, including serum PSA levels at diagnosis (*n* = 269) and overall survival time after treatment with first‐line ARSI (enzalutamide or abiraterone acetate, *n* = 99).

### 2.2 Sample preparation and microarray analysis

Fresh‐frozen bone metastasis samples were cryo‐sectioned into extraction tubes, and RNA was isolated using the AllPrep DNA/RNA/Protein method (Qiagen, Hilden, Germany). The percentage of tumor cells in the samples was determined by morphological examination of parallel sections stained with hematoxylin‐eosin. The RNA quality was ensured by Bioanalyzer evaluation (Agilent Technologies Inc., Palo Alto, CA) and cDNA was generated from 100 ng RNA, using the GeneChip TM WT PLUS Reagent Kit (Thermo Fisher Scientific Inc., Life Technologies, Carlsbad, CA, USA). Clariom D Human Arrays were hybridized, washed, stained, and scanned according to manual, using the GeneChip TM Fluidics Station 450 and the GeneChip TM Scanner 3000 7G. R (v4.0.5), and package oligo (v1.52.1) was used to preprocess the bead‐level data. Raw data were normalized by the Robust Multi‐Array Average (RMA) algorithm (function rma).

### 2.3 Classification of the metastasis subtypes MetA‐C

The MetA‐C subtypes were determined by unsupervised cluster analysis of 72 bone metastasis samples (GSE29650 and GSE101607, https://www.ncbi.nlm.nih.gov/geo/), as previously described [17]. The top 60 differentiating gene products per subtype were identified, defined by the lowest *P*‐values in Mann–Whitney *U* test and a median fold change ≥ 1.5 separating one subtype from the others. From the top 180 MetA‐C differentiating genes, 157 transcripts were further selected as consistently MetA‐C associated (Table S2) based on principal component analysis (PCA) of transcript profiles of the PC metastasis samples analyzed in [5] and [7].

The publicly available *in silico* tool CIBERSORT [18] was used to estimate the MetA‐C fractions of each metastasis sample. To enable this, representative triplicate reference samples were selected per subtype out of the pool of the original 72 bone metastasis [17], as determined by the PCA cluster output described above. Data corresponding to the robustly MetA‐C‐associated 157 transcripts were compiled for the triplicate samples into a reference sample file and in turn used to construct a subtype‐specific gene expression signature, using default CIBERSORT parameters, including quantile normalization. RMA signal values for the Clariom D samples, normalized RNA‐Seq counts for the Quigley samples [5], and fragments per kilobase of transcript per million mapped reads for the Abida samples [7] were used for estimating the MetA‐C fractions. For RNA‐Seq data [5, 7], quantile normalization was disabled, as per CIBERSORT recommendation.

### 2.4 Differential expression and gene set enrichment analyses

Differential expression analysis was performed using R (v4.0.5) package limma (v3.46.0). Samples were stratified based on their dominant (highest) subtype fraction comparing each subtype against the rest. T‐statistics from the resulting tables were used to generate preranked gene lists for each of the subtypes. Gene set enrichment analysis (GSEA) was performed using the public The gsea Software (v4.1.0) to run GSEAPreranked, for quantifying enrichment of hallmark gene sets (*n* = 50), acquired from the Molecular Signatures Database (MSigDB) collect (v7.4). Normalized enrichment scores (NES) were used as a metric to compare phenotypes in between the MetA‐C subtypes.

### 2.5 Androgen receptor activity, proliferation, and neuroendocrine‐like scores

As previously described [21], metastasis samples were given individual AR activity and proliferation scores assigned as the first principal component t1‐values obtained by PCA of relative transcript levels predefined to be associated with canonical AR activity [19] and cell cycle activity (proliferation) [10], respectively. Similarly, individual NEPC scores were obtained by PCA of predefined NEPC‐associated gene transcript levels [20].

### 2.6 DNA analysis and modeling of MetA‐C‐differentiating gene aberrations

Of all single nucleotide variants (SNVs) reported in [5] and [7], the frameshift insertions/deletions, nonframeshift insertions/deletions, startloss, stopgain, stoploss, splice variants, and nonsynonymous SNVs predicted to be deleterious by SIFT and PolyPhen were retained. Mutated genes with a frequency ≥ 0.02 in each cohort, respectively, were selected for downstream analysis. For each gene, a Mann–Whitney *U* test was performed to check for difference in MetA, B, or C fraction levels between samples harboring an SNV and samples with no SNV in the specific gene.

For analysis of copy number variation (CNV), all coding genes were retained and called as having a gain if the copy number was ≥ 4 and loss if the copy number was ≤ 1. Genes were kept in downstream analyses if they had an RNA count ≠ 0 in > 97% of the samples and had a CNV in ≥ 3% of the samples. Remaining genes were tested for difference in MetA, B, or C fraction levels and RNA levels between samples harboring an CNV and samples with no CNV in the specific gene using Mann–Whitney *U* tests. Genes showing significant differences (*P* < 0.05) in both tests were selected for modeling using orthogonal partial least squares discriminant analysis (OPLS‐DA) based on corresponding RNA counts.

## 3 Results

### 3.1 Prostate cancer metastases are heterogeneous for the MetA‐C subtypes

**Fig. 1:**
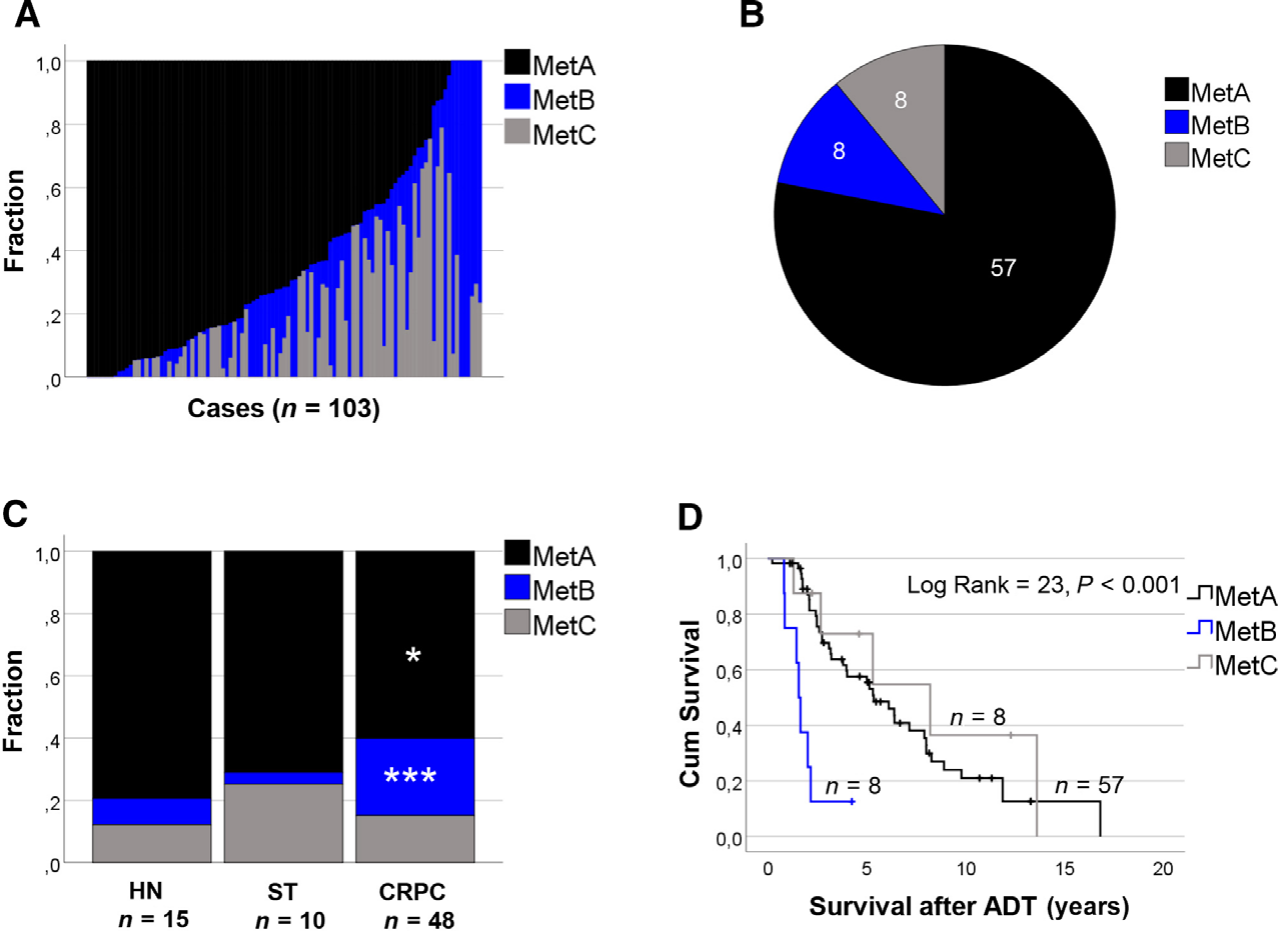
The MetA‐C subtypes in relation to biological and clinical characteristics of metastasis samples profiled by Clariom D array analysis. (A) Estimated fractions of MetA‐C in 103 metastasis samples from 67 patients (for details, Table S1). (B) Numbers of metastasis samples classified as MetA‐C, respectively, based on their dominating subtype estimate: MetA (*n* = 57), MetB (*n* = 8), MetC (*n* = 8). (C) The mean fraction estimates of MetA‐C in metastasis samples of hormone‐naïve (HN, *n* = 15), short‐term castrated (ST, *n* = 10), and castration‐resistant prostate cancer (CRPC, *n* = 48) patients. ***P < 0.001 and *P < 0.05 in comparison with the HN group, according to the Mann–Whitney *U* test. (D) The MetA‐C subtypes (*n* = 57, 8, and 8, respectively) in relation to cancer‐specific survival after ADT, according to Kaplan–Meier survival analysis. From 6 patients with CRPC, metastasis samples were collected at two time‐points with additional therapies given in‐between. Those patients are represented twice in clinical analyses, explaining the total of 73 samples shown in B–D.

The MetA‐C content of each metastasis sample analyzed by Clariom D array analysis was estimated, based on its expression levels of 157 predefined MetA‐C differentiating gene transcripts using the CIBERSORT tool [18]. Most metastasis samples showed a heterogeneous MetA‐C subtype (Fig. 1A). The tumor content of MetA was inversely correlated to the tumor fractions of MetB and MetC (*Rs* = −0.68, *P = *1.9E‐11 and *Rs* = −0.58, *P = *1.8E‐8, respectively, *n* = 103), while the MetB and MetC showed no clear relationship (*Rs* = −0.089, *P = *0.37, *n* = 103). Replicate samples obtained from the same metastasis showed high concurrence in the estimated fractions of MetA‐C (Table S3), and patients were represented by the mean MetA‐C estimates in further analysis. In analysis of MetA, MetB, and MetC in relation to clinical characteristics, six patients with metastasis samples collected at two different time‐points, and with therapy for CRPC given in‐between, were represented twice (Table S4), leaving a total of 73 metastases for further analysis. Those 73 metastasis samples showed a MetA‐C distribution of 78, 11, and 11% (Fig. 1B), when classified based on their dominating subtypes.

### 3.2 The MetB subtype is enriched in castration‐resistant metastases and associated with poor prognosis

The CIBERSORT‐estimated distribution of metastasis subtypes was compared with respect to previous patient treatment. The estimated fraction of MetB was found to be significantly higher (mean fraction 25 vs. 8.5%, *P = *0.00072), and that of MetA significantly lower (mean fraction 60 vs. 79%, *P = *0.010), in CRPC compared with hormone‐naive cases (Fig. 1C). Accordingly, patients with a dominating MetB subtype showed the poorest prognosis after ADT, with a median survival time of only 1.6 years in comparison with 5.3 and 8.2 years for patients with MetA and MetC, respectively (*P = *1.3E‐5, Fig. 1D). In Cox survival analysis, the MetB subtype was associated with increased risk of cancer‐related death after ADT, while, in contrast, the MetA subtype was associated with reduced risk (Table 2). The fraction of MetB provided independent prognostic information from MetA and from available clinical variables (Table 2).

**Table 2:** Estimated fractions of the metastasis subtypes A‐C (MetA‐C)^a^ in Clariom D‐profiled samples (*n* = 73) in relation to tumor and patient characteristics.

	Correlation analysis			Cox survival analysis			
	MetA	MetB	MetC	Univariate^b^		Multivariate^b^	
				HR	*P*	HR	*P*
MetA	1	−0.69**	−0.60**	0.36	0.050	2.1	0.36
MetB	−0.69**	1	−0.046	16	0.000071	21	0.022
MetC	−0.60**	−0.046	1	0.58	0.47		
Age (dia)	−0.042	−0.10	0.11	1.04	0.045	1.05	0.034
Age (sampling)	−0.10	0.09	0.08	1.0	0.082		
Serum PSA (dia)	0.37**	−0.51**	−0.033	1.0	0.32		
Serum PSA (sampling)	0.46**	−0.43**	−0.22	1.0	0.52		
AR activity score^a^	0.76**	−0.27*	−0.68**	0.86	0.091		
Proliferation score^a^	−0.29*	0.77**	−0.41**	1.1	0.020	1.00	0.94
NEPC‐like score^a^	−0.56**	0.12	0.57**	1.1	0.053		
Epithelium (%)	0.17	−0.004	−0.27*	0.72	1.0		

**P < 0.01. *P < 0.05 (Spearman correlation).

^a^ The MetA‐C content, and androgen receptor (AR) activity, proliferation, and neuroendocrine prostate cancer (NEPC)‐like scores were determined as described in the materials and methods section.

^b^ All variables were analyzed as continuous variables in survival analysis.

The estimated fraction of the MetA subtype within a metastasis sample was positively correlated to the patient serum PSA level measured at diagnosis (*Rs* = 0.40, *P = *0.0019) and at metastasis sampling (*Rs* = 0.46, *P = *8.1E‐5), while the fraction of MetB showed an inverse relation to serum PSA levels (*Rs* = −0.51, *P = *7.0E‐6; *Rs* = −0.43, *P = *2.4E‐4) (Table 2). The MetA‐C subtypes were not obviously related to Gleason score at diagnosis, primary tumor treatment (results not shown) or patient age (Table 2). A weak inverse relationship was observed between the MetC content and the fraction of tumor epithelial cells in the metastasis samples (*Rs* = −0.27, *P = *0.026) (Table 2).

There was no relation observed between the metastasis subtypes and previous treatment of CRPC with AR targeting therapies (bicalutamide, enzalutamide, abiraterone acetate) or chemotherapy (Table 3). However, the metastasis samples from patients who had been treated with osteoclast inhibiting agents (zoledronic acid or denosumab) contained very low MetB fractions in comparison with other samples (median values 3 vs. 23%, *P = *0.011, Table 3). In contrast, the median MetA fraction was significantly higher in zoledronic acid/denosumab‐treated compared to untreated metastases (88 vs. 60%, *P = *0.024, Table 3).

**Table 3:** Estimated fraction of the metastasis subtypes A‐C (MetA‐C)^a^ in Clariom D‐profiled samples in relation to previous treatment for castration‐resistant prostate cancer.

	MetA (%)	MetB (%)	MetC (%)
Bicalutamide			
No, *n* = 18	60 (37; 84)	29 (7.0; 46)	3.5 (0; 16)
Yes, *n* = 30	72 (41; 87)	16 (5.0; 28)	11 (0; 35)
Abiraterone acetate			
No, *n* = 40	63 (37; 85)	18 (7.0; 40)	8.0 (0; 22)
Yes, *n* = 8	74 (49; 91)	12 (1.5; 24)	8.0 (1.5; 26)
Enzalutamide			
No, *n* = 43	57 (37; 86)	16 (7.0; 38)	5.0 (0; 28)
Yes, *n* = 5	72 (63; 81)	16 (5.0; 21)	14 (14; 16)
Chemotherapy			
No, *n* = 29	57 (36; 84)	24 (7.0; 39)	9.0 (0; 29)
Yes, *n* = 19	72 (55; 85)	14 (4.5; 26)	7.9 (0; 18)
Ra223			
No, *n* = 45	63 (37; 83)	20 (7.0; 36)	9.0 (0; 26)
Yes, *n* = 3	84 (84; 91)	2.0 (1.0; 8.0)	3.0 (1.5; 9.5)
Zoledronic acid/Denosumab			
No, *n* = 42	60 (37; 83)	23 (8.0; 39)	9.5 (0; 26)
Yes, *n* = 6	88 (83; 98)*	3.0 (0.0; 14)*	4.0 (0; 17)

Continuous variables given as median (25th; 75th percentiles), *P < 0.05; **P < 0.01.

^a^ The MetA‐C fractions were determined as described in the materials and methods section.

^b^ Castration therapy included surgical ablation, LHRH/GnRH agonist therapy, or bicalutamide treatment.

^c^ Castration therapy for 1 day to 3 months before metastasis surgery.

### 3.3 The MetB subtype is enriched in liver metastases and associated with poor prognosis in external patient cohorts

The content of MetA‐C was estimated also in external metastasis samples profiled by RNA sequencing within the Quigley (*n* = 99) and the Abida (*n* = 332) cohorts and evaluated in relation to available clinical data [5, 7, 9]. The Abida cohort was further divided into two subcohorts, based on samples sequenced with the polyA (*n* = 266) or capture (*n* = 208) libraries. All three cohorts gave results that were in good agreement with results from the Clariom D cohort, as specified below. In addition, they provided the possibility of evaluating metastasis content of the MetA‐C subtypes in relation to metastatic site.

**Fig. 2:**
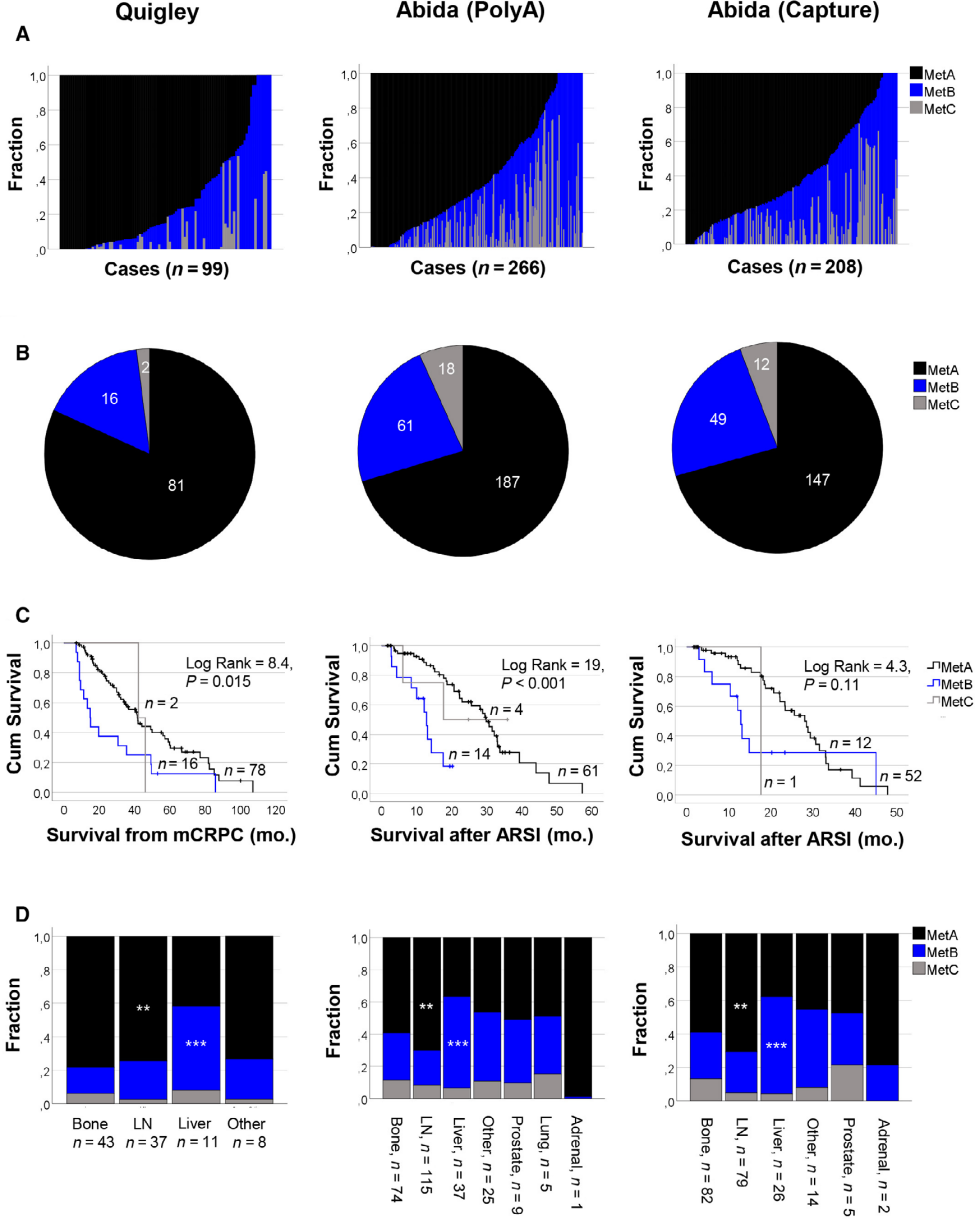
The MetA‐C subtypes in relation to biological and clinical characteristics of metastasis samples from different patient cohorts profiled by RNA sequencing. (A) Estimated fractions of MetA‐C per metastasis sample in the Quigley (*n* = 99) and Abida (PolyA library, *n* = 266) and Abida (Capture library, *n* = 208). (B) Numbers of metastasis samples classified as MetA, B, and C, respectively, based on their dominating subtype estimate in the Quigley (*n* = 81, 16, and 2), Abida PolyA (*n* = 187, 61, and 18), and Abida Capture (*n* = 147, 49, and 12) cohorts. (C) The MetA‐C subtypes in relation to cancer‐specific survival after diagnosis of mCRPC or after treatment with AR signaling inhibitors (ARSI), respectively, according to Kaplan–Meier survival analysis. Analysis included a total of 96, 79 and 65 patients in the Quigley, Abida Poly A, and Abida Capture cohorts, respectively, with available follow‐up data. (D) The mean fraction estimates of MetA‐C in 99 (Quigley), 266 (Abida PolyA), and 208 (Abida Capture) metastasis samples, respectively, in relation to metastatic sites. ***P < 0.001 and **P < 0.01 in comparison with bone metastases, according to the Mann–Whitney *U* test.

The Quigley and Abida metastases showed clear heterogeneity for the MetA‐C (Fig. 2A) and inverse relationships between the MetA and MetB as well as the MetA and MetC subtypes (Table S5). When classified based on the dominating metastasis content (Fig. 2B), patients with MetB metastases showed poor prognosis compared to other patients, expressed as overall survival after mCRPC diagnosis (Quigley cohort) or after treatment with first‐line ARSI (Abida cohorts) (Fig. 2C). Interestingly, all three cohorts showed significant enrichment of the MetB subtype in liver metastases and of the MetA subtype in lymph node metastases (Fig. 2D).

The estimated content of MetA‐C showed no relation to former treatment with taxanes or enzalutamide/abiraterone acetate in the cohorts examined (results not shown).

### 3.4 The MetA‐C subtypes show differences in tumor biology

**Fig. 3:**
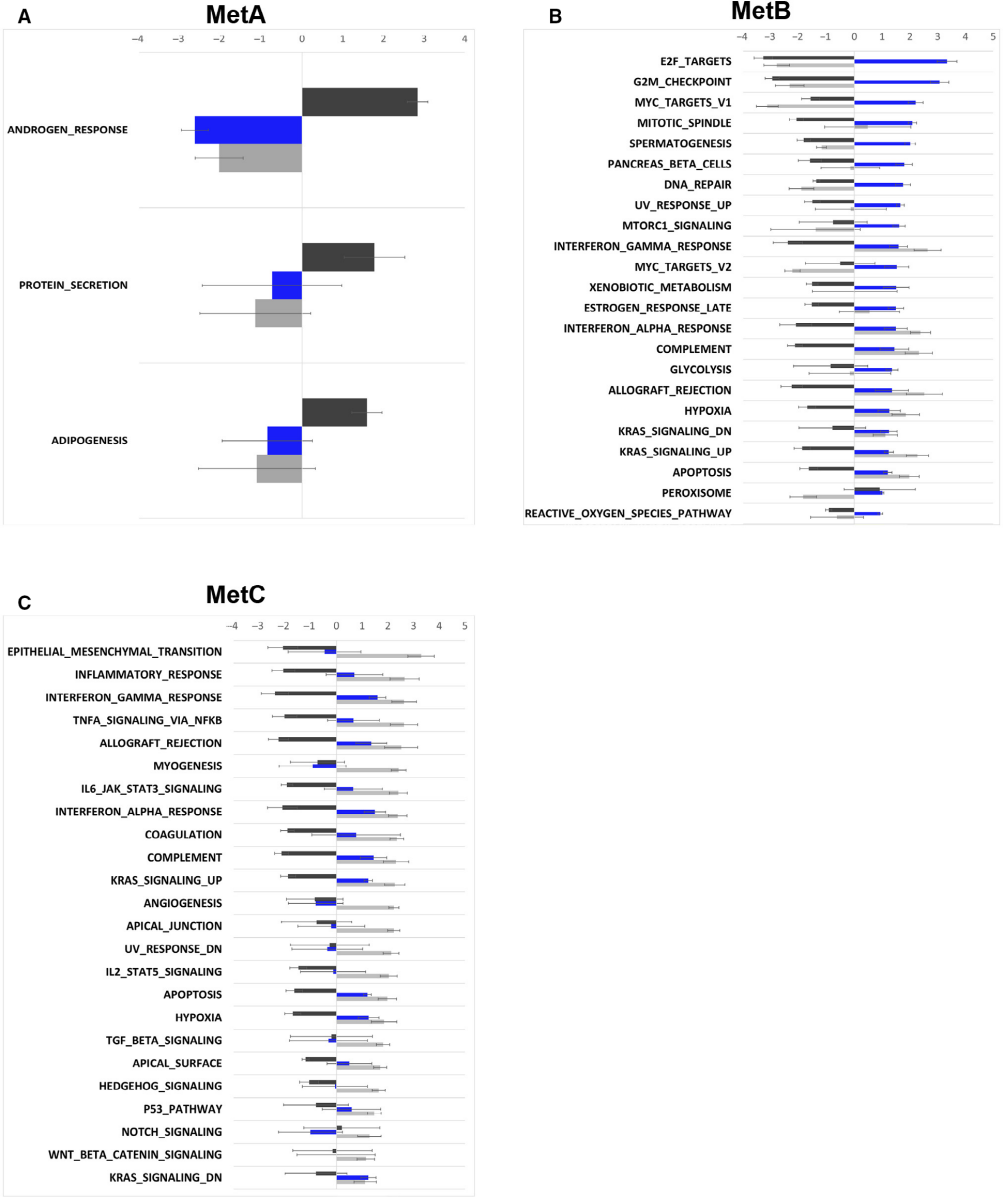
Enriched hallmark gene sets in the MetA‐C subtypes. Hallmark gene sets consistently enriched in metastasis samples classified as MetA (A), MetB (B), or MetC (C) in four separate metastasis sample cohorts (Clariom D, Quigley, Abida polyA, and Abida capture) are shown, based on significant NES. Samples were classified based on their dominating subtype estimate.

To explore biological differences between the MetA‐C subtypes, GSEA was performed, based on the dominating subtype of each sample. Enrichment of hallmark gene sets was analyzed for each subtype in comparison with the other two in all cohorts examined (Table S6), and hallmarks being consistently enriched with significant NES in MetA, MetB, or MetC samples were notified. The MetA phenotype showed high androgen response, protein secretion, and adipogenesis compared to MetB and MetC (Fig. 3A), while the MetB phenotype was characterized by many hallmarks associated with cell cycle activity and DNA repair (Fig. 3B). The MetC phenotype clearly showed signs of epithelial‐to‐mesenchymal transition (EMT), myogenesis, and angiogenesis in comparison with both MetA and MetB, while many enriched hallmarks associated with inflammatory responses were shared with the MetB subtype (Fig. 3C).

To further examine important biological differences between the MetA‐C subtypes, each metastasis samples were assigned scores for AR activity, proliferation, and NEPC‐like features, based on their relative expression levels of predefined gene sets [10, 19, 20]. As shown in Table 2 for the Clariom D‐profiled metastases, the content of MetA was significantly correlated to the AR activity score (*Rs* = 0.76, *P = *6.2E‐15), while it was inversely correlated to the proliferation (*Rs* = −0.29, *P = *0.011) and NEPC scores (*Rs* = −0.56, *P = *2.2E‐7). In contrast, the MetB and MetC estimates showed inverse correlations with the metastasis AR activity scores (*Rs* = −0.27, *P = *0.021 and *Rs* = −0.68, *P = *4.4E‐11, respectively) (Table 2). Increasing MetB content was associated with higher proliferation score (*Rs* = 0.77, *P = *2.4E‐15), while increasing MetC content was associated with decreasing proliferation score (*Rs* = −0.41, *P = *3.3E‐4) and increasing NEPC score (*Rs* = 0.57, *P = *1.5E‐7) (Table 2). Similar relationships between the MetA‐C content of metastasis samples and their corresponding AR activity, proliferation, and NEPC‐like scores were observed within the Quigley and the Abida cohorts (Table S5).

### 3.5 The MetA‐C subtypes in relation to genomic tumor aberrations, and their independent prognostic value

The estimated MetA‐C content of the metastasis samples of the validation cohorts was evaluated in relation to reported ETS gene fusions [5, 7], gain and loss of coding gene regions, and SNVs predicted to be deleterious. For CNVs, only gains and losses that significantly implied on their respective RNA transcript levels were considered to have possible impact on the metastasis subtype.

**Fig. 4:**
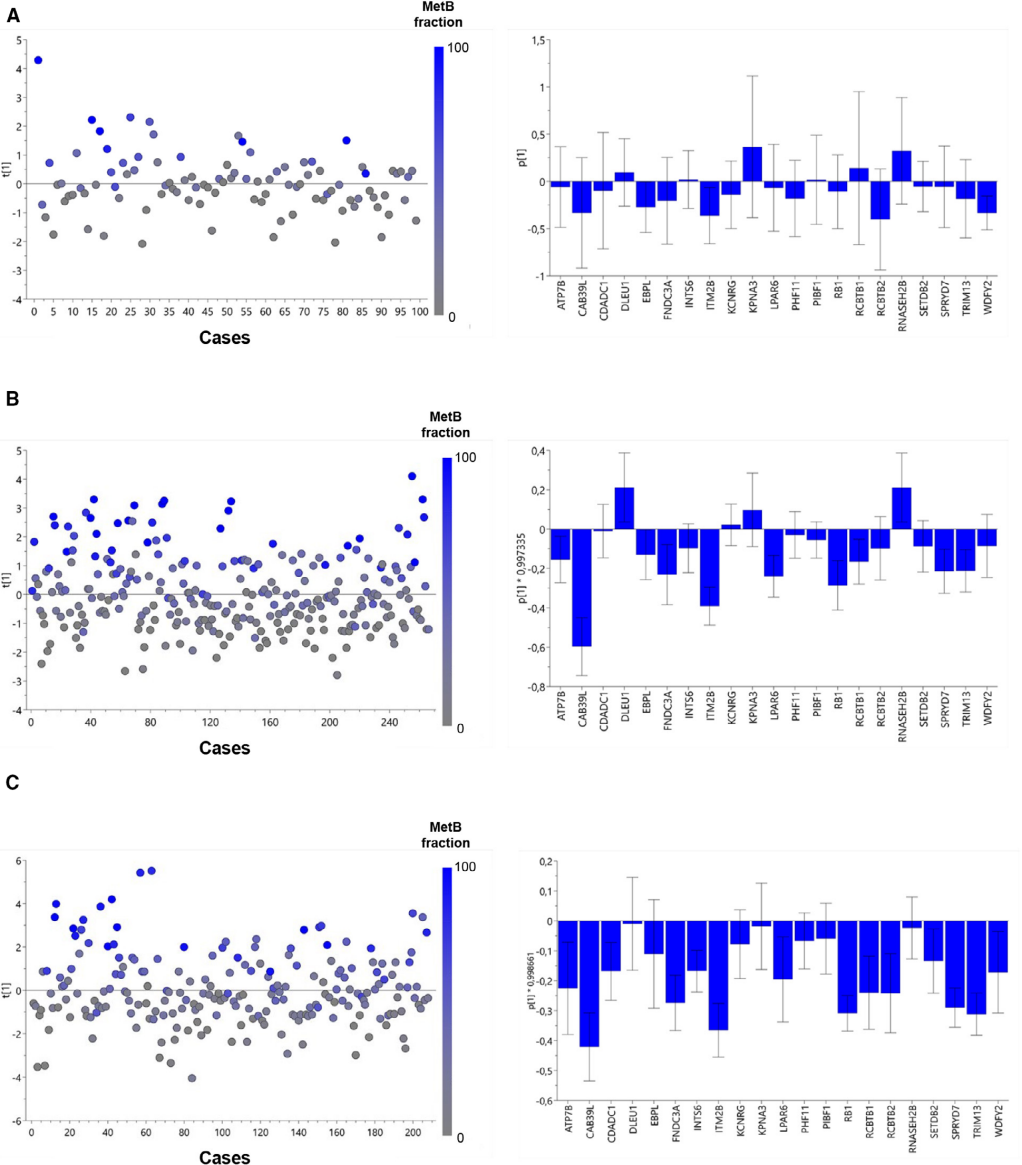
Modeling of tumor fraction of the MetB subtype, based on transcript levels of genes showing systematic associations between CNV loss and reduced expression. Orthogonal partial least squares discriminant analysis (OPLS‐DA) of metastasis samples showing score and loading plots for the Quigley (A), Abida polyA (B), and Abida capture (C) cohorts, based on transcript levels for 21 genes on chromosome 13 in relation to the estimated sample fraction of MetB. The 21 genes were selected based on significant associations between CNV loss, transcript reduction, and increased metastasis content of MetB, consistently observed in all cohorts examined.

In the Abida subcohorts, metastasis samples positive for ETS gene fusions (predominantly TMPRSS2‐ERG) showed significantly higher MetA content than the negative cases, while no clear association between the MetA‐C content and ETS gene fusion status was observed in the Quigley cohort (Fig. S1). The MetB subtype systematically showed CNV losses and corresponding reductions in transcript levels of 21 genes on chromosome 13, including *RB1* (Fig. 4), while the MetA and MetC subtypes showed no consistent CNVs (Table S7). The MetB‐associated CNVs were further analyzed by OPLS‐DA based on their corresponding transcript levels to model their impact on the sample subtype (Fig. 4). Overall, low expression levels of *ITM2B* were strongly associated with the MetB subtype in all three cohorts. In the Abida cohorts, reduced levels of also other transcript levels corresponding to the 21 CNV losses showed clear impact on the MetB subtype, including *RB1* (Fig. 4B–C). Diverse SNVs were associated with the MetA‐C subtypes in the Quigley and Abida cohorts (Suppl. Fig. S2), but only *RB1* was consistently found being associated with MetB.

Altogether, aberrations in *RB1* (deleterious SNVs and/or CNV loss) were observed in 63% and 18‐31% of the metastases with a dominant MetB subtype in the Quigley and Abida data, respectively. As previously reported, *RB1* aberrations were associated with poor patient prognosis after mCRPC diagnosis (Quigley cohort [9]) and after treatment with first‐line ARSI (Abida cohorts, [7]), but importantly, the MetA‐C subclassification consistently provided independent prognostic information to those aberrations (Table 4).

**Table 4:** Cox regression analysis of the metastasis subtypes A‐C (MetA‐C) and *RB1* status in relation to overall survival after androgen receptor signaling inhibition (Abida cohorts) or diagnosis of metastatic castration‐resistant prostate cancer (Quigley cohort). All variables were analyzed as categorical variables in survival analysis. The MetA‐C class was defined based on the dominating tumor subtype per sample. SNVs, single nucleotide variants; CNV, copy number variation.

Abida, capture library (*n* = 65)	HR	*P*
Univariate		
*RB1* (SNVs and/or CNV loss), *n* = 11	3.5	0.0028
Multivariate		
Subtype		
MetA, *n* = 52	1	
MetB, *n* = 12	2.3	0.041
MetC, *n* = 1	1.0	0.97
*RB1* (SNVs and/or CNV loss), *n* = 11	3.9	0.0020

Abida, polyA library (*n* = 76)	HR	*P*
*RB1* (SNVs and/or CNV loss), *n* = 16	1.9	0.098
Multivariate		
Subtype		
MetA, *n* = 58	1	
MetB, *n* = 14	4.9	0.00023
MetC, *n* = 4	1.0	0.95
*RB1* (SNVs and/or CNV loss), *n* = 16	1.6	0.21

Quigley (*n* = 95)	HR	*P*
Univariate		
*RB1* (SNVs and/or CNV loss), *n* = 18	2.4	0.006
Multivariate		
Subtype		
MetA, *n* = 77	1	
MetB, *n* = 16	2.0	0.022
MetC, *n* = 2	1.5	0.59
*RB1* (SNVs and/or CNV loss)	2.0	0.03

## 4 Discussion

By analysis of 676 metastasis tissue samples originating from four patient cohorts, this study verifies the clinical relevance of classifying PC metastases into the transcriptomic subtypes MetA‐C [17]. By estimating the metastasis content of MetA‐C, PC patients with very poor prognosis (MetB) can be differentiated from patients with relatively favorable prognosis (MetA) after primary ADT and subsequent treatment for CRPC. The study also verifies consistent biological and genetic differences between the MetA‐C subtypes that could be used to improve treatment of patients with metastatic PC.

Most metastases were estimated to be heterogeneous for MetA‐C subtypes, with MetA being most frequently observed. If this heterogeneity represents a mixture of tumor epithelial cell clones with different characteristics, as have been previously demonstrated for primary PC [15, 16], or if the heterogeneity represents single tumor clones showing subtype characteristics in‐between more pure MetA‐C tumor clones, is not known and need to be explored using methods with resolution at the single cell level.

The MetA‐C frequencies observed here were comparable to those originally reported [17]. The content of MetB was higher in CRPC compared to hormone‐naïve cases, indicating that castration therapy selects for the MetB subtype. This may explain the somewhat higher frequencies of MetB seen in the external cohorts (Quigley, Abida), exclusively comprised by CRPC samples, in comparison with the metastases profiled in the current study (Clariom D) that also included 21% treatment‐naïve and 14% short‐term castrated metastases. Thus, it is possible that the MetA‐C subtypes are plastic and transform during ADT. To examine this hypothesis, metastasis samples need to be longitudinally collected and analyzed from individual patients during therapy. Furthermore, parallel analysis of metastases and corresponding primary tumors is needed to evaluate to what extent the MetA‐C subtypes are intrinsic, and if the metastasis subtype can be predicted by analyzing the primary tumor, as previously suggested [17].

The MetA‐C subtypes were originally identified in bone metastases [17]. Here, they were detected also at other metastatic sites, although at different frequencies, suggesting that the MetA‐C subtypes have tropism for different organs, similar to what have been previously reported for breast cancer subtypes [22]. Liver metastases consistently showed high MetB content in comparison with bone metastases. As liver metastases frequently develop in patients with NEPC and as treatment with second‐generation ARSI seems to drive the development of a NEPC‐like phenotype with *RB1* and *P53* aberrations [23], we were surprised to find no enrichment of MetB in patients treated with enzalutamide, abiraterone acetate, or other treatments for CRPC.

The GSEA confirmed the biological characteristics of MetA‐C originally described [17], with MetA showing a dominating androgen response, MetB showing cell cycle activities and DNA repair, and MetC showing EMT together with other diverse activities in then tumor microenvironment, including myogenesis, angiogenesis, and inflammatory responses. In addition, both the MetB and MetC subtypes showed NEPC‐like characteristics. While the transcripts associated with AR activity in the MetA subtype and the transcripts associated with cell cycle activity in the MetB subtype most probably originate from the tumor cells, the main characteristics described for the MetC subtype may be related to activities in the metastatic stromal cells. Potential drivers of the MetC phenotype and its abundant stroma still need to be identified.

The MetA‐C metastasis subtypes show similarities with, but are not identical to [17], the transcriptomic‐based subtypes PCS1‐3 described for primary prostate tumors [13] and the luminal A, B, and basal subtypes originally described for primary breast cancer, but applicable also on primary PC [14, 24]. The role for transcriptomic subtyping of primary PC still needs to be proven for metastatic patients, although a recent paper showed that patients with Luminal B and higher Decipher risk primary PC had benefit from receiving docetaxel in addition to ADT as first‐line systemic therapy for metastatic disease [25]. Also, the origin of transcriptomic metastasis subtypes needs to be clarified; do they arise from diverse cell types or do they develop over time due to genetic and/or epigenetic clonal expansions influenced by the microenvironment and/or by therapy?

Recently, we identified a DNA methylation signature associated with AR activity in PC bone metastasis samples [21]. High promoter methylation levels in canonically AR‐regulated/regulating genes were observed in MetB and MetC cases. Together with the current findings, demonstrating associations between MetA‐C and diverse genetic aberrations, this may provide clues for improved therapy of metastatic PC. Patients with the MetA subtype would probably benefit from early add‐on therapies maximally targeting AR signaling, such as abiraterone acetate, enzalutamide, darolutamide, and others under development [26]. Patients with the MetB subtype, on the other hand, showing androgen independence, high proliferation, and frequent *RB1* aberrations, would probably benefit from early chemotherapy and treatment with drugs currently being suggested for NEPC, such as cell cycle inhibitors and epigenic modulators [27, 28]. Given their high tumor DNA repair response, MetB patients might also be responsive to PARP inhibitors [28, 29], and the reduced MetB fraction seen after treatment with zoledronic acid indicates sensitivity to bone‐targeting therapies. The MetC subtype may hypothetically benefit from treatment with immunomodulating drugs, based on the predicted high inflammatory response, or by other drugs targeting processes in the microenvironment, such as angiogenesis inhibitors. If, however, most metastases are heterogeneously composed by different tumor subclones with varying responses to treatments, many patients would probably benefit from tailored treatment combinations.

## 5 Conclusions

**Fig. 5:**
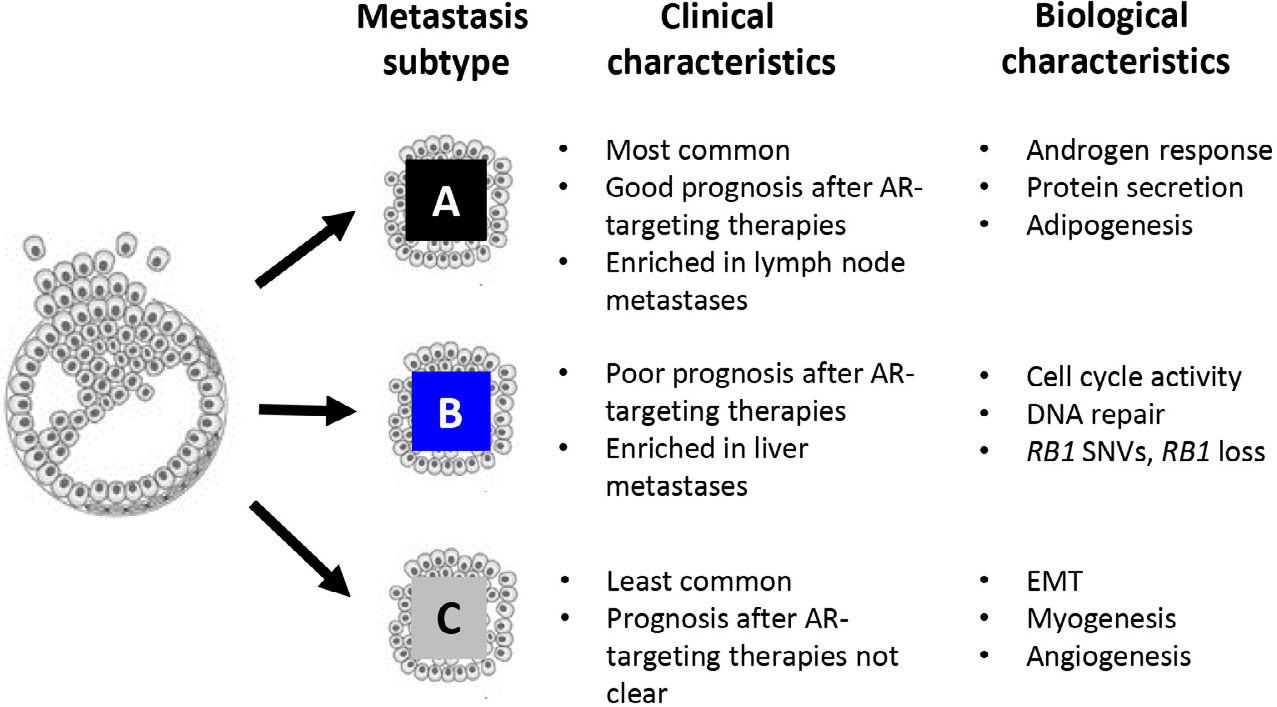
Clinical and biological characteristics of the transcriptomic‐based MetA‐C subtypes. Figure shows clinical and biological characteristics of the specified subtypes verified by the current study. Outstanding questions still to be answered: (i) are the MetA‐C subtypes intrinsic and traceable back to the primary tumor? (ii) are the MetA‐C subtypes plastic and influenced by differences in the microenvironment or by treatments? (iii) does tumor MetA‐C heterogeneity reflect mixed cell clones or cells with intermediate subtypes? (iv) how do MetA‐C cells interact with other cell types in their environment? SNVs, single nucleotide variants; EMT, epithelial‐to‐mesenchymal transition.

PC metastases can be differentiated into clinically relevant subtypes, based on distinct transcriptomic profiles. The MetA‐C subtypes show different biology, organ tropism, and prognosis after AR targeting therapies (Fig. 5). Phenotypic, genetic, and epigenetic characteristics of MetA‐C suggest diverse therapies for metastatic PC to be tested in relation to subtype. Interpretation of the results is limited by the retrospective design of the study, and the therapy‐predictive value of the MetA‐C subtypes needs to be evaluated in prospective settings. Furthermore, MetA‐C subtype‐specific treatment strategies should be developed, primarily to improve survival for MetB patients.

## Conflict of interest

ET, AB, and PW have a pending patent application (‘Methods for diagnosis and prognosis of prostate cancer’, EP2020/054682). The other authors declare no conflict of interest.

## Author contributions

ET, LK, JN, and PW conceived and designed the study. LK, JS, HJ, ML, SC, and CTK acquired the data. ET, LK, JS, EF, and PW analyzed and interpreted the data. PW wrote the manuscript draft that all authors critically revised. CTK, JED, AW, SC, AJ, KW, and AB contributed with technical and materiel support. AB and PW supervised the study.

### Peer review

The peer review history for this article is available at https://publons.com/publon/10.1002/1878‐0261.13158.

## Supporting information


**Fig S1**. Associations between the metastasis subtypes A‐C (MetA‐C) and ETS gene fusion status.

**Fig S2**. Associations between the metastasis subtypes A‐C (MetA‐C) and deleterious single nucleotide variants (SNVs).

**Table S1**. Patient and sample characteristics.

**Table S2**. Gene transcripts used for classification of the metastasis subtypes A‐C (MetA‐C).

**Table S3**. Estimated fractions of the metastasis subtypes A‐C (MetA‐C) in replicate samples collected from the same metastatic site at one time‐point.

**Table S4**. Estimated fractions of the metastasis subtypes A‐C (MetA‐C) in samples collected from the same patient at two different time‐points.

**Table S5**. Estimated fractions of the metastasis subtypes subtypes A‐C (MetA‐C) in relation to tumor and patient characteristics of the Abida polyA, Abida capture, and Quigley metastasis cohorts.

**Table S6**. Hallmarks being consistently enriched in the metastasis subtypes A‐C (MetA‐C), respectively, in the Clariom D, Abida capture, Abida polyA, and Quigley metastasis cohorts.

**Table S7**. Copy number variation (CNV) gains and losses of coding regions in relation to corresponding transcript levels and estimated fractions of metastasis subtypes A‐C (MetA‐C) in samples of the Quigley, Abida polyA and Abida capture cohorts.

## Data Availability

The data generated during the study is openly available in NCBI's Gene Expression Omnibus at [https://www.ncbi.nlm.nih.gov/geo/], reference number [GSE189343]. Data corresponding to the Abida cohort are publicly available as described in [7]. Data corresponding to the Quigley cohort were made available as described in [5] and [9] and through personal contact with the corresponding author.
