# Correction to: Clinical and biological relevance of the transcriptomic‐based prostate cancer metastasis subtypes MetA‐C

**DOI:** 10.1002/1878-0261.13579

**Published:** 2024-01-24

In the manuscript by Elin Thysell *et al*. [1], Figure legends for Supplementary Fig. S1 and Fig. S2 and the Supplementary file ‘Supplementary information_rev’ were missing but are now provided.

Figure legends for Supplementary Fig. S1 and Fig. S2 should read as below:

**Figure S1.** Associations between the metastasis subtypes A‐C (MetA‐C) and ETS gene fusion status. Mean fraction estimates of the MetA‐C are shown in relation to the ETS gene fusion status of samples in the Quigley (A), Abida polyA (B), and Abida capture (C) cohorts. ***P < 0.001, **P < 0.01, and *P < 0.05, according to the Mann–Whitney *U*‐test.

**Figure S2.** Associations between the metastasis subtypes A‐C (MetA‐C) and deleterious single nucleotide variants (SNVs). Heatmaps are showing deleterious SNVs significantly associated with the estimated fraction of MetA (black), MetB (blue), or MetC (gray) in metastasis samples of the Quigley (A), Abida capture (B), and Abida polyA (C) cohorts. Samples are sorted by increased fraction of MetB.

In Supplementary Table 1, in rows 63 and 64 the anatomical site for two samples (unique sample IDs 61 and 62) has been changed from crista to LN.

On page 847 in the Material and Methods section 2.1. Patient samples, the sentence ‘Core biopsies from the iliac crest or lymph node metastases were obtained from 16 and 2 patients, respectively’. should have read ‘Core biopsies from the iliac crest or lymph node metastases were obtained from 14 and 4 patients, respectively’.

The correction of these errors has not altered the interpretation of data and did not affect the conclusions presented in the article.

The authors apologize for any inconvenience caused.
