# Supplementary figures and images for: Clinical and biological relevance of the transcriptomic‐based prostate cancer metastasis subtypes MetA‐C
Source: Mol Oncol. 2021 Dec 27;16(4):846–59. doi: 10.1002/1878-0261.13158 (PMC8847984; doi:10.1002/1878-0261.13158)

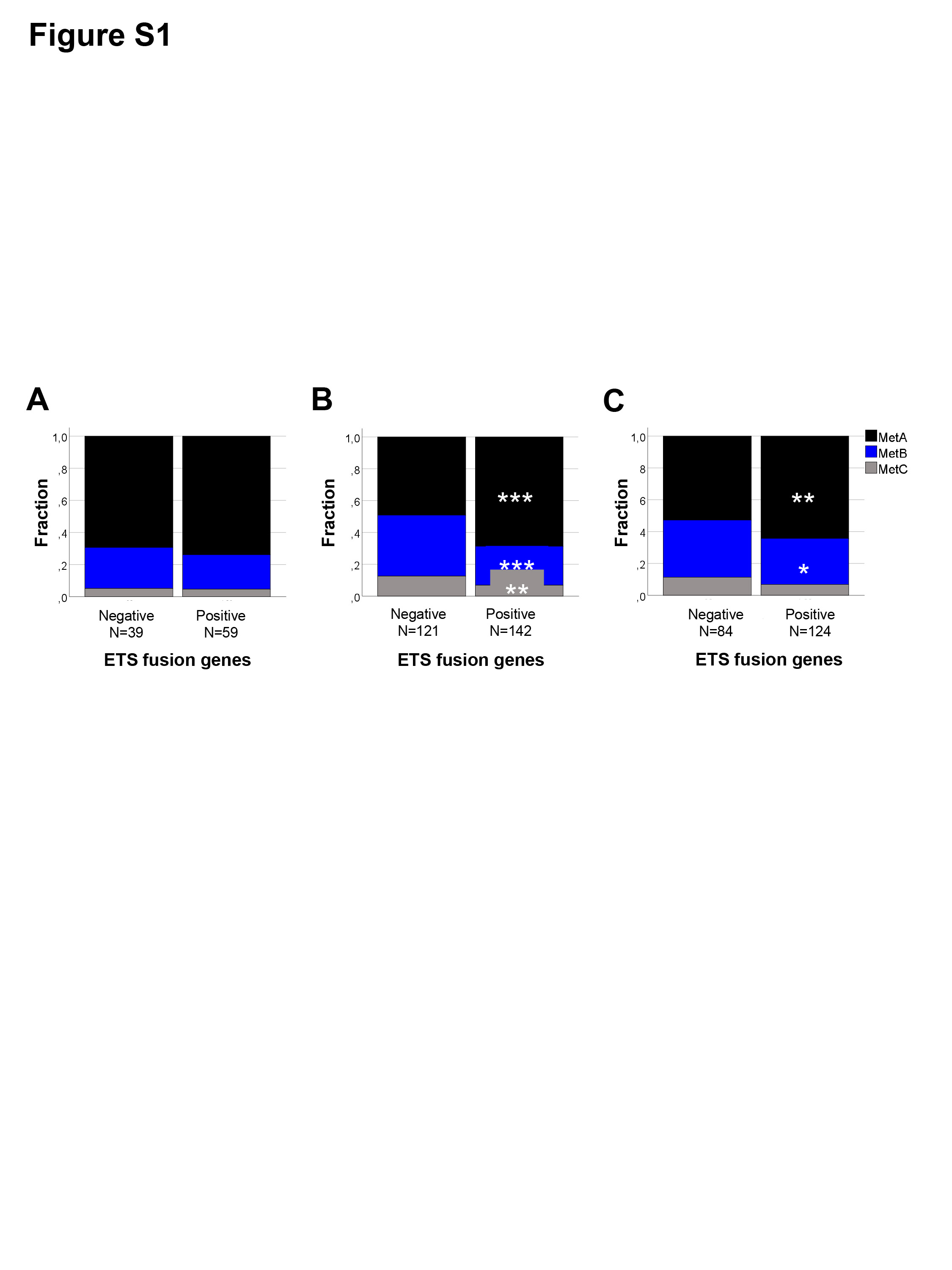

Supplement: Supplementary file 1 — Fig S1. Associations between the metastasis subtypes A‐C (MetA‐C) and ETS gene fusion status. [file MOL2-16-846-s001.jpg]

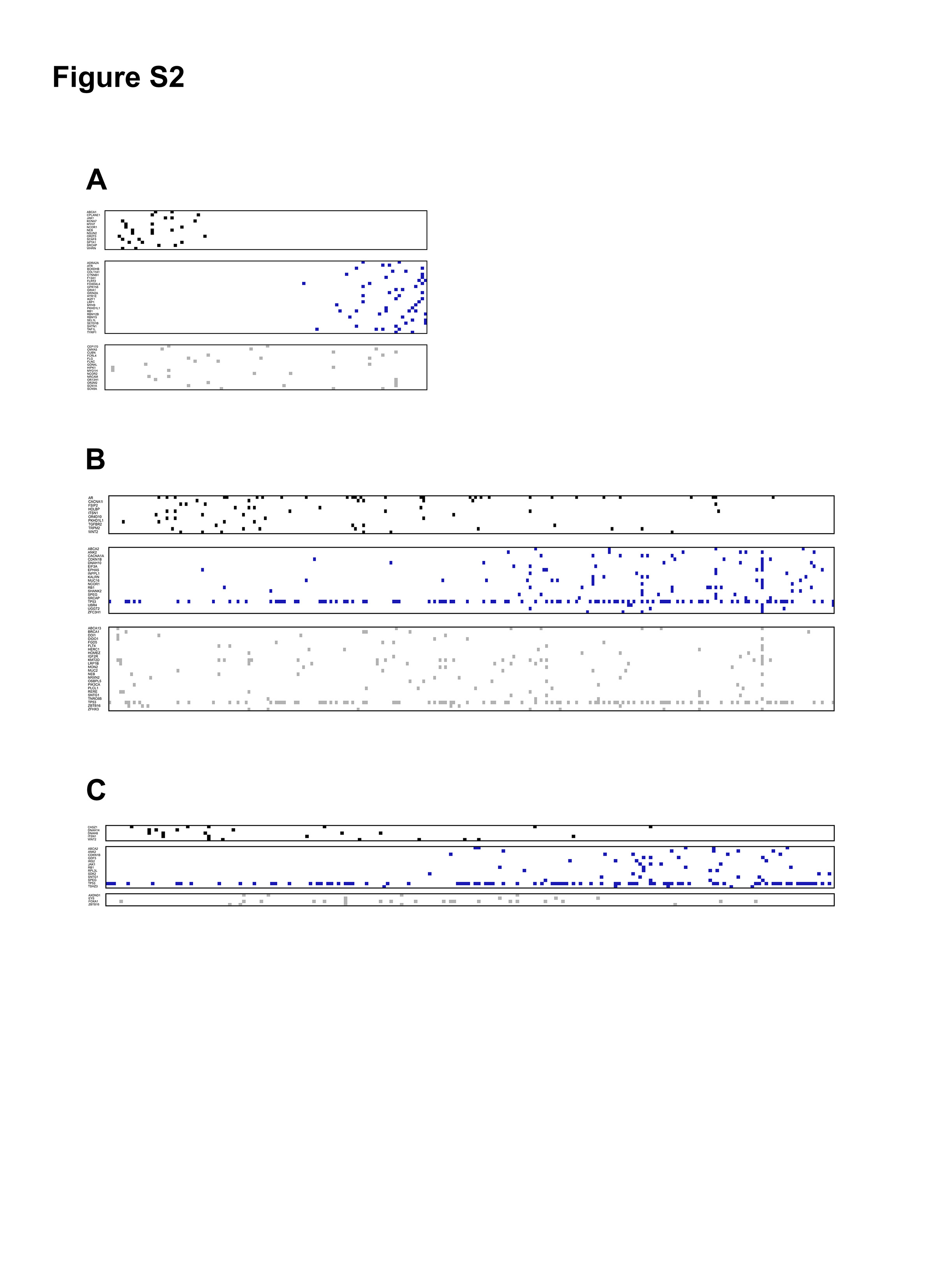

Supplement: Supplementary file 2 — Fig S2. Associations between the metastasis subtypes A‐C (MetA‐C) and deleterious single nucleotide variants (SNVs). [file MOL2-16-846-s007.jpg]
